# An ultra wideband-high spatial resolution-compact electric field sensor based on Lab-on-Fiber technology

**DOI:** 10.1038/s41598-019-44644-y

**Published:** 2019-05-30

**Authors:** V. Calero, M. -A. Suarez, R. Salut, F. Baida, A. Caspar, F. Behague, N. Courjal, L Galtier, L. Gillette, L. Duvillaret, G. Gaborit, M. -P. Bernal

**Affiliations:** 1 FEMTO-ST Institute, UMR 6174, CNRS, 15Bis Avenue des Montboucons, Besançon, 25030 France; 2 KAPTEOS SAS, 354 Voie Magellan, Sainte-Helene-du-lac, 73800 France

**Keywords:** Optics and photonics, Medical research

## Abstract

Non-intrusive, wide bandwidth and spatial resolution are terms often heard in electric field sensing. Despite of the fact that conventional electromagnetic field probes (EMF) can exhibit notable functional performances, they fail in terms of perturbation of the E-field due to their loaded metallic structure. In addition, even though electro-optical technology offers an alternative, it requires large interaction lenghts which severely limit the sensing performances in terms of bandwidth and spatial resolution. Here, we focus on miniaturizing the interaction volume, photon lifetime and device footprint by taking advantage of the combination of lithium niobate (LN), Lab-on-Fiber technologies and photonic crystals (PhC). We demonstrate the operation of an all-dielectric E-field sensor whose ultra-compact footprint is inscribed in a 125 μm-diameter circle with an interaction area smaller than 19 μm × 19 μm and light propagation length of 700 nm. This submicrometer length provides outstanding bandwidth flatness, in addition to be promising for frequency detection beyond the THz. Moreover, the minituarization also provides unique features such as spatial resolution under 10 μm and minimal perturbation to the E-field, accompanied by great linearity with respect to the E-field strength. All these specifications, summarized to the high versatibility of Lab-on-Fiber technology, lead to a revolutionary and novel fibered E-field sensor which can be adapted to a broad range of applications in the fields of telecommunications, health and military.

## Introduction

Electromagnetic field (EMF) sensing is critical for applications as varied as telecommunications, high power and frequency pulse detection^1^, electromagnetic compatibility, optimization of the electrical behavior of the devices^2^ and brain or other tissues monitoring for healthcare applications^3,4^. Traditional EMF probes are based on conductive materials^5^ which, in addition to scatter the E-field to be measured, might produce electrical coupling with the source or surrounding elements. Despite the fact that open-ended waveguide (OEWG) technology stands as a promising alternative, the bandwidth to be measured is limited to the size, leading to over-meter long devices for sensing low frequencies^6^. Electro-optical (EO) technologies have arised in the last decades since they hold the use of ferroelectric materials which modulate an optical carrier as a function of the applied E-field, getting rid of electric coupling and EM inducted currents which compromise the integrity of the sensors^1^. This galvanic isolation allows the generation of devices that minimally perturb the measuring E-field and that are robust to intense pulses, in addition to present wide bandwidth extending from 1 *Hz* to decades of GHz^7^.

Most of the existing E-field sensors based on ferroelectric materials employ Mach Zehnder (MZ) or polarization state modulation schemes based on EO-crystals such as bismuth germanium oxide (BGO), potassium dihydrogen phosphate (KDP), zinc telluride (ZnTe) or lithium niobate (LN)^7,8^. However, the use of these bulk crystals often requires large sensing lengths, in the order of milimeters, which sets a limitation in terms of frequency bandwidth, spatial resolution and E-field perturbation^6^. For this reason, the achievement of reduced figures of merit for light-matter interaction-based applications becomes a key issue that needs to be solved. One promising way consists of using Photonic Crystal (PhC) structures which perform slow-light and phase matching mechanisms for the enhancement of the light-matter interaction^9,10^. Recently, the potential of PhC structures has been demonstrated in different ferroelectric materials for the achievement of enhanced non-linear (NL) and electro-optical (EO) performances^11–13^. In most of the cases the PhCs are based on micro- and nano-meter dielectric waveguides^14–17^ which are potential candiates in terms of scaling the photonic chip density. However, for the design of compact E-field sensing heads, waveguides lead to unnecessary propagation volumes and complicated light coupling schemes^18^. A reflection based scheme can considerably reduce the device footprint, whilst the direct coupling into the photonic structure may reduce the coupling losses by employing an optical fiber attached to the sensor head. Several compact E-field sensors based on this idea have been demonstrated^19,20^ where a bulk EO-crystal is attached to a fiber tip, demonstrating the potential of fiber-tip reflection based schemes.

In this work, we go beyond the miniaturization of the sensor head by combining Lab-on-Fiber (LOF) technology. Indeed, the intrinsic properties of optical fibers to transport a microscopic spot of light to a small and recondite location when coupled to the properties of electro-optical photonic crystal sensor make a new group of LOF that can be used for elecric field sensing applications that were not possible up to now.

The idea underlying LOF technology is to transform a standard optical fiber into a compact sensor by integrating at the optical fiber (typically the apex) functionnalized materials and/or optically sensitive components of nanometric dimensions (the lab part)^21–24^. LOF has been successfully used for instance as a radiation dosimeter for ultra-high dose monitoring^25^, as a label-free chemical and biological sensor^26^, and as *in situ* monitoring of drug release^27^.

In this work, the lab part will combine an EO material with PhC geometries for novel applications in electric field sensing. Indeed, thanks to the LOF configuration, the measure of the electric field may by physically very close (few micrometers), take place in a harsh environment and be very localized (few squared micrometers).

By doing so a nano-patterned sub-micrometer thin film located at the tip of a fiber can provide a sensing volume compact enough to achieve a bandwidth in the order of decades of THz^19^ and a spatial resolution comparable with the mode field diameter (MFD) of the supporting fiber (10.5 μm for the SMF-28e). In addition, the choice of lithium niobate (LN) as sensitive material allows its application in harsh and corrosive environments due to the stability of its optical properties and favorable chemical conditions. These features make possible the most compact all-dielectric E-field sensor ever reported in terms of interaction volume, but it is also its miniaturized device footprint that reveals its potential to provide outstanding performances in terms of EM invisibility, ultra-high spatial resolution, THz bandwidth, stability to chemicals, high temperature and intense fields through a versatile and ultra-lightweight sensing head device.

## Design and Simulations

### Design of the structure

**Figure 1:**
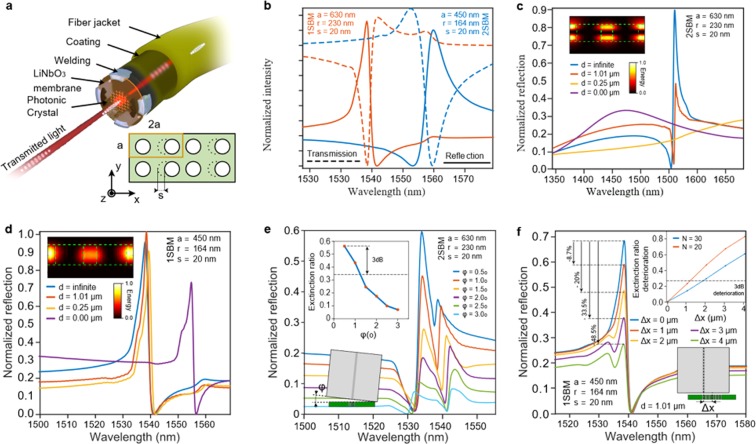
Sketch and numerical analysis of the fiber tip sensor. **(a**) Rendered image of the PCS attached to the fiber extremity (top) Geometry of the photonic crystal (bottom). (**b**) Transmission and reflection spectra for both 1SBM and 2SBM operating structures. (**c**,**d**) Computed reflection spectra in function of the air/gap length between the fiber section and the PCS for both (**c**) 2SBM and (**d**) 1SBM. In the inset, its respective electric field distributions. (**e**,**f**) Tolerance analysis to (**e**) the unacuraccy on the parallelism between the fiber section and the PCS and (**f**) the miss-centering error.

The schematic concept of the E-field sensor is illustrated in Fig. 1(a). An ultra-thin and monolithic air-coated thin membrane of LN is located on the facet of a single mode fiber (SMF). This configuration is known as Photonic Crystal Slab (PCS) since an air-filled planar PhC is designed within the membrane. The PhC lattice is mainly based on a square lattice defined by the period *a* and hole radius *r*, as depicted in the inset of Fig. 1(a). However, the unit cell is extended by introducing an asymmetry by shifting the odd holes a distance *s* towards the x-axis every two columns, producing a band folding phenomenon^28^. This alteration of the band diagram leads to guided modes (GM) that can sometimes be efficiently coupled to the out-of-plane modes thanks to the phase matching provided by the periodic structure^29–31^. This phenomenon, known as Guided Resonance (GR), provides a strong light localisation in addition to an enhanced vertical confinement due to the high refractive index contrast^32,33^. Moreover, the GRs scheme provides outstanding spectral performances in terms of spectral slope due to the presence of an anomalous interference pattern known as the Fano phenomenon^34^.

Since PCS are periodical structures, light behavior within the lattice is described by Bloch Modes. Each mode is characterized by its resonant wavelength *λ*_*r*_ which is generally proportional to the lattice parameters *a* and *r* for non-dispersive materials^35^. In our case, and due to the band-folding phenomenon, two Slow Bloch Modes (SBM) arise for a propagation along the *γ* direction (i.e. normal incidence) as shown in^36^. The fact that a wide variety of equipment operates at the telecom wavelength (*λ*_*r*_ at 1550 nm) presents an advantage since the structural parameters can be rescaled to set the resonant wavelength *λ*_*r*_. Thus, the structural parameters lead to *a* = 630 *nm*, *r* = 230 *nm* and *a* = 450 *nm*, *r* = 164 *nm* for the two first SBMs^36^, denoted as 2SBM and 1SBM, respectively. Their computed reflection spectra of the finite photonic structure are respectively represented by the continous blue and red lines in Fig. 1(b), which theoretically demonstrate the high coupling efficiency that GRs provides. The low frequency SBMs generally present a stronger light localization within the sensing media^28^ that has been theoretically estimated to produce an enhancement of the EO performances above 400 times with respect to bulk LN^36^. Furthermore, this effect may produce high sensitivity to geometrical^37^ or surrounding media changes^38^, which makes the GRs more susceptible to geometrical imperfections induced by the PhC fabrication, but also to any refractive index changes within the GRs near field, i.e. an optical fiber tip. The fabrication of the LN-based PCS is optimized and assessed in a recent paper^39^. However, concerning the integration onto the fiber tip, the fiber may alter the SBMs. Furthermore, the rigidity of the LN slab and the impossibility to apply batch processes for the PhC fabrication^40^ leads to restrictions on the PCS size and flexibility, in contrast with Si or polymer based structures^41–43^, which strongly impacts on the integration tolerances.

### Study of the fiber integration factors

In order to study the feasibility of the integration onto the fiber tip, the mentioned constraints are studied by Finite Difference Time Domain (FDTD) simulations whose configuration is described in the methods section. The influence of the fiber on the SBMs is studied by considering the dielectric isolation between the slab and the fiber facet. The resulting reflection spectrum due to an air gap introduced between both volumes is presented on Fig. 1(c,d). Indeed, the proximity of the fiber induces a drastic degradation of the spectral performances for the 2SBM. This is, however, not observed for the 1SBM. The steady field distribution of the 2SBMs, represented in the inset, reveals its even mode nature defined by a node in the middle of the slab. Consequently, the electric field is more localized in the interface regions, increasing its interaction with the surrounding media. In contrast, the 1SBM localizes most of the energy in the middle of the slab, which makes it less sensitive to the fiber positioning.

The implementation of the 2SBM requires a minimum air gap for dielectric isolation, which may complicate the assembly and can also deteriorate the parallelism between both surfaces. This is investigated by introducing an angle *ϕ* between the fiber facet and the PCS interface. Figure 1(e) determines a maximum tolerance of *ϕ* = 1.5°, revealing the feasibility of the 1SBM since the slab can be directly in contact with the fiber tip, ensuring *ϕ* to be negligible. Therefore, the 1SBM lattice parameters and the number of periods *N* can be assessed as a compromise solution between the PCS-fabrication time, performed by serial processes, and GRs coupling performances. Moreover, accordingly with Fig. 1(f), lower PhC areas induce critical tolerances of the miscentering with respect to the fiber core center. Specifically, a structure with *N* = 42 leads to a PhC area of 19 μm × 19 μm and allows miss-centerings up to 2 μm since it presents a deterioration of the spectral aspect ratio under 3 *dB*, which is technologically feasible.

## Fabrication and Optical Characterization

### Fabrication of the fiber-tip sensor

**Figure 2:**
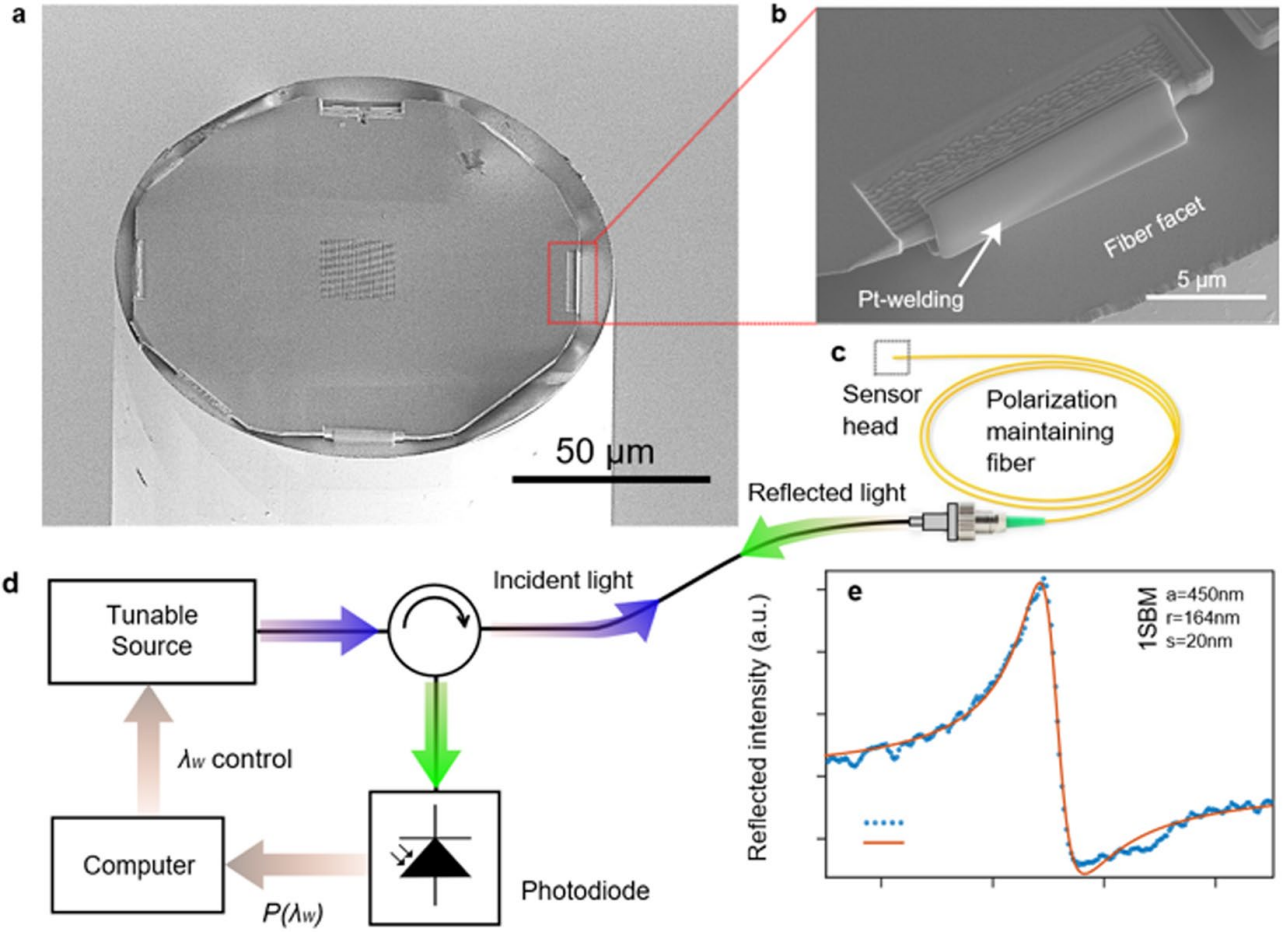
Assembly and test of the Fano sensor **(a**,**b**) SEM images of the (**a**) assembled structure and (**b**) one of the point weldings between the fiber and the membrane. (**c**) Macroscopic view of the fibered sensor. (**d**) Measuring system accompanying the sensor head. (**e)** Measured reflection spectrum of the assembled PCS.

The structure is fabricated from a lithium niobate on insulator (LNOI) stack wafer manufactured by ion slicing techniques^44,45^. During this work, four Fano sensor heads were successfully fabricated. The Fano resonance wavelength error was of 18 nm at most in the membrane and of 40 nm once integrated onto the fiber. The fabrication procedure of the free-standing LN membrane and the PCS within is beyond the scope of this paper, but investigated in a previous work^39^. The integration of the PCS on the fiber facet is performed by the point welding method reported by Solgaard group^46^. Thus, we use a dual beam device, FEI Helios Nano Lab600i in order to deposit the weldings, which is assisted by a micromanipulator, Kleindiek MM3A EM for the membrane positioning. Since the GRs used are polarization dependent, the sensor is based on a polarization maintaining (PM) fiber, whose birefringence is induced by panda-type elements since it presents reduced losses in comparison with other PM topologies, optimizing the signal-to-noise ratio (SNR). The adapted point welding method employed for the integration of the LN-based PCS is detailed in the Methods section. The result from the integration process leads to the Fano-based PCS sensor probe shown in Fig. 2(a–c). The head consists of an octogonal 700 nm thick membrane inscribed in a circle whose diameter is 125 μm. One of the weldings based on Pt employed for the bonding is shown in Fig. 2(b). By analyzing the SEM images we can estimate that the miss-centering error between the PhC and the fiber centers to be under 2 μm and both surfaces are observed to be quasi-parallel.

### Optical characterization

The reflection spectra is characterized by employing the system sketched in Fig. 2(d). It consists of a tunable continuous wave (CW) source, YENISTA TUNICS T100S, plugged to an optical circulator. The system is entirely based on panda-type PM fibers in order to reduce the optical losses by mode matching with the sensor fiber. The reflected light is detected by a photodiode whose data is collected by a computer that also controls the source wavelength tuning. The assembled structure leads to the reflection spectrum represented by the blue tightened dots in Fig. 2(e) and the resonant wavelength is located at 1576 nm. The resonance presents an average spectral slope of 4.96 *dB*/*nm*, showing better spectral performances than the 2SBM^39^, as it is a lower frequency mode in addition to be the highest ever reported on LN-PCS^40,47–49^.

## Electro-Optical Performances

The versatility of the system depicted in Fig. 2(d) allows us to directly use it for E-field sensing. In order to create an electric field, the sensor head is placed between two coplanar electrodes, which are directly fed by a frequency synthetizer. The experimental acquision of the modulation is performed using a network analyser tuned at the same frequency of the applied E-field. Its frequency and span are optimized to ensure a noise floor around −125 *dBm*.

### Evaluation of the optimal working point

**Figure 3:**
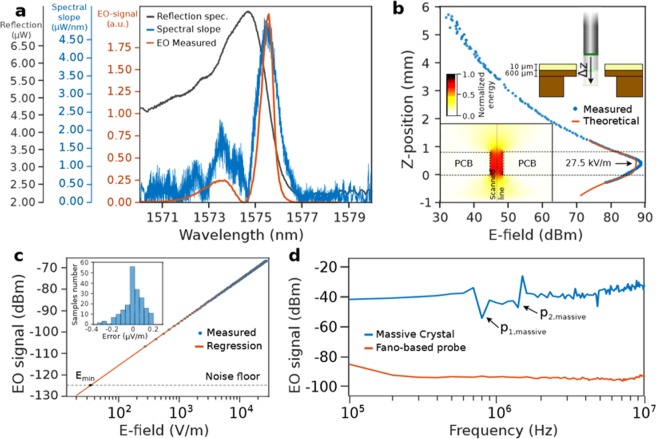
Electro-optical performances of the E-field sensor probe. **(a**) Theoretical intensity spectrum of the Fano resonance (black) represented with his slope value (blue) and the EO-modulation strength in function of the laser source wavelength (red). (**b**) Calibration of the sensor by fitting the distribution of the E-field along Z. In the inset, the computed E-field distribution produced by the electrode PCB. (**c**) EO-modulation strength with respect to the applied E-field. In the inset, the statistical distribution of the measurement error relatively to the fitting curve. (**d**) EO-modulation in function of the E-field frequency for (blue) a massive crystal sensor^19^ and (red) the Fano sensor.

The EO modulation relies on the fact that any applied electric field along the crystallographic Z-axis introduces a linear variation of the refractive index with respect to the E-field. This modification of the refractive index leads to a shift of the Fano resonance frequency Δ*λ*_*r*_. Thus, considering a working point wavelength *λ*_*w*_ set within the resonance slope, the external applied electric field will modify the reflected optical power. The EO modulation strength is proportional to the spectral slope, e.g. the absolute value of the first derivative of the optical spectral response, illustrated by the blue curve in Fig. 3(a), revealing the spectral advantages of the geometry due to the pronounced spectral slope.

This relation can be observed on the red line in Fig. 3(a) which represents the EO modulation strength as the working point wavelength *λ*_*w*_ is varied between 1570 nm and 1580 nm. Good agrement of the EO modulation strength with the spectral slope (blue curve) is demonstrated since the position of the peaks and their spectral width are quasi identical. In addition, the difference of the modulation strength of both peaks reveals the asymmetrical features of Fano resonances. These results demonstrate that the magnitude of the modulation induced by EO effect is linear with the derivative of the reflection spectrum, putting in evidence the Fano resonance as the origin of the modulation. These peaks determine the inflection points that, setting the working point at the maximum modulation strength and the greatest linearity with respect to the applied E-field are ensured. Therefore, *λ*_*w*_ is fixed at the inflection point at *λ*_*w*_ = 1575.6 *nm* whose EO modulated signal spectrum demonstrates the optimal EO-modulation with an strength that exceeds the 30 *dB* above the noise floor.

### Calibration of the sensor

The calibration is performed by fitting the measurements with the computed E-field resulting from Finite Element Method (FEM) simulations, represented at the lower inset of Fig. 3(b). The printed circuit board (PCB), where the coplanar lines are located, presents a hole to allow the probe head to sweep along the z-axis as sketched at the upper inset of Fig. 3(b). Both computed and measured curves are shown in Fig. 3(b), where good agreement is observed. The maximum E-field value is 27.5 *kV*/*m*, which is therefore employed to test the linearity and RF performances.

### Sensitivity and linearity with respect to the electric field

The linearity has been assessed by tuning the output voltage of the frequency synthetiser from 50 V_pp_ down to the minimum allowable value by the system, 20 mV_pp_. In Fig. 3(c), the measured data is well fitted through linear regression, giving the proportional link *α* = 125.67 μV/(V/m) between the E-field to be measured and the output signal. The standard deviation of the error between the measurement and the linear fitting curve indicates an E-field strength dispersion of *σ*_*E*_ = 0.077 V/m, revealing the linearity provided by Fano lineshapes in comparison with traditional approaches. Moreover, the distribution of the error is centered at 0 ($\bar{\varepsilon }$ = 7 × 10^−16^), thus validating the absence of instabilities of the sensor during the experiment. Note that both, E-field strength and EO-signal values are represented using a logarithmic scale in order to extract the actual minimum detectable field. The minimum detectable field is determined by the intersection between the noise floor and the linear fitting curve, delimiting the E-field detection threshold to 32 V m^−1^ ${{\rm{Hz}}}^{-\frac{1}{2}}$. This detection threshold reveals unique features since, despite of the fact that bulk crystals can offer a threshold of ${\rm{m}}V{{\rm{m}}}^{-1}H{z}^{-\frac{1}{2}}$, the PCS strongly increases the light-matter interaction, allowing such small structures to detect electric fields.

### RF-spectral performances

The advantages of the minituarization appear also on the available bandwidth. In order to detect different RF signals with the same sensor head, the response of the device in the presence of RF fields must be invariant with respect to the frequency, which is difficult on bulk-crystals based devices since the piezoelectric resonances modulate its EO-coefficient *r*_33_(*f*) due to the macroscopic interaction length^50^. As represented by the blue curve in Fig. 3(d), the first piezo resonance is located at *p*_1_ = 780 *kHz* for a crystal length of a few milimeters^19^. In contrast, the spectrum of the Fano-based sensor, represented by the red line in Fig. 3(d), presents a significant RF flatness up to 10 *MHz* since the 700 *nm* interaction length shifts up the frequency at which piezo resonances occur.

### Vectorial selectivity

**Figure 4:**
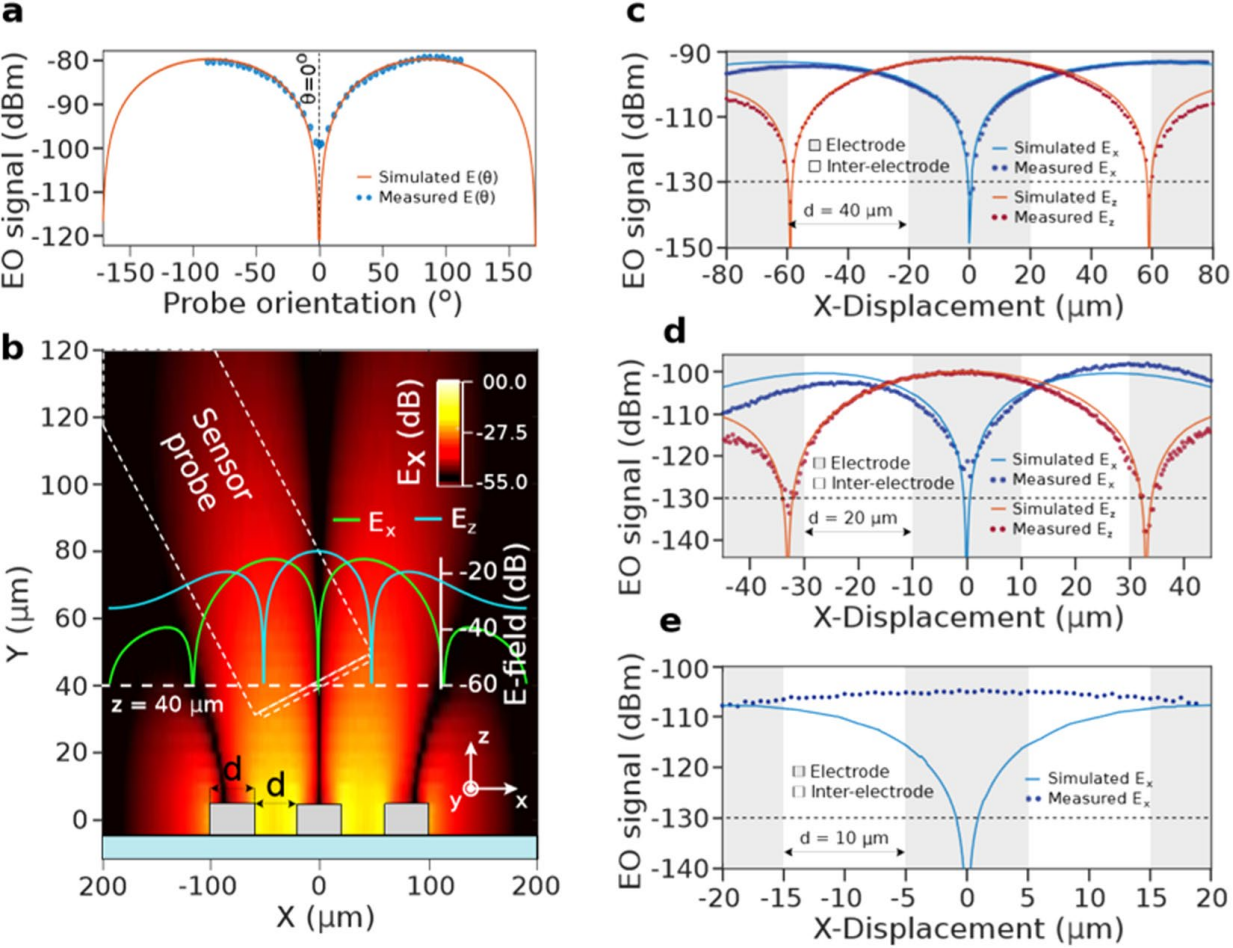
Mapping-related EO performances. **(a**) EO-modulation with respect to the angle divergence between the crystalline extraordinary axis of LN and the E-field. (**b**) FEM-computed Electric field distribution produced by the coplanar lines. The green and blue curves represent respectively the *E*_*x*_ and *E*_*z*_ components along the x-axis at a distance of 40 away from the lines. (**c–e**) 1-D mapping along the x-axis at a distance of 40 μm for the *E*_*x*_ (blue) and *E*_*z*_ components (red), dots correspond to the measurement and the curve to the FEM simulated E-field for the interelectrode distances of (**c**) 40 μm, (**d**) 20 μm and (**e**) 10 μm.

An important feature of an E-field sensor relies on the ability to perform vectorial measurements, i.e. to be sensitive to one E-field vector component while rejecting the transverse ones. The selectivity has been assessed using coplanar electrodes. The photonic crystal sensor is placed normal and in between the electrode plane. Thus, the electric field is present at the x-component and is zero at the y-component (as shown in Fig. 4). The photonic crystal is rotated following the fiber revolution axis between the electrodes around the optical wave vector direction for angles ranging from $-\frac{\pi }{2}$ to $\frac{\pi }{2}$. The experimental data are shown in Fig. 4(a) (blue dots) and is well fitted with an ideal sinusoidal response, exhibiting a good agreement and estimating a vectorial selectivity greater than 20 *dB*.

### Spatial resolution and electric field mapping

One of the main advantages of the GRs based sensor is its ability to confine the light into a small space. As the E-field measurement can be conceived as the convolution between the field itself and the optical mode within the PCS, the sensor may be able to resolve features under 20 μm.

In order to evaluate this, three coplanar electrodes are designed on a glass substrate as depicted in Fig. 4(b), where the central one is plugged to the frequency synthetizer whilst the other ones to the electrical ground. The fabrication procedure is detailed in the Methods section. The lines are parallel to the y-axis accordingly with the axis reference in Fig. 4(b). In the same figure, the FEM computed *E*_*x*_ field distribution is represented along the Z plane. Both components, *E*_*x*_ and *E*_*z*_, corresponding to a distance of 40 μm away from the electrodes, are respectively plotted by the red and light blue curves. *E*_*x*_ presents its zero at *x* = 0, whilst *E*_*z*_ presents two zeros at both sides. As the interelectrode distance is decreased, the aspect ratio of the E-field distribution increases enough so that the sensor is not able to measure the E-field minima due to its wide integration area.

To verify this, the sensor is translated along the x-axis and tilted around 15° to avoid parasitical reflections from the substrate while keeping the PhC y-axis parallel to the E-field component to be measured. The sensing of both *E*_*x*_ and *E*_*y*_ along the x-axis is represented in Fig. 4(c–e) by blue and red dots, respectively. The probe detects properly the zeros for *d* = 40 μm located at *x* = −59, 0 and 59 μm as the EO-signal is under the noise floor, in accordance with the computed E-field represented by the solid curves. For *d* = 20 μm, the zeros are clearly determined, however the EO-signal at *x* = 0 is about −125 *dBm*, not under the noise floor, revealing the first limitation of the integration area. Indeed, when decreasing *d* to 10 μm, see Fig. 4(e), the sensor is not able to discriminate the zero at the origin, revealing the spatial resolution limit to be between 20 and 10 μm which proves the potential for the ultra-compact GR of the PCS structure.

## Discussion

We have studied the behavior of the GRs in the presence of a fiber which could interact with its near-fields. This has revealed that the light distribution within the slab plays an important role since modes which present most of the light located at the interfaces are more vulnerable to the fiber proximity. We have determined a structure that allows the direct integration of the PCS on the facet getting rid of any air gap in between which may complicate the integration procedure. The point welding method, which already presents a notable control of the different integration parameters, becomes feasible for the integration of such tiny PhC structures. Indeed, we have succesfully proven the integration succesfully onto the fiber facet and fully characterized by achieving a resonant wavelength reproducibility of ±40 *nm* and a spectral slope of 4.96 *dB*/*nm*, the highest ever achieved on LN-based PCS. It is its outstanding spectral performances provided by the Fano phenomenon, added to the light-matter interaction enhancement gives by the GRs, which makes possible to demonstrate the EO-modulation in such a compact interaction length. A great linearity has been experimentally determined with respect to the E-field, thanks to the Fano spectral slope, and an extended flatness of the detection bandwidth in comparison to bulk crystal devices has been observed. This is possible because the ultra-short cavity length shifts up the piezo-electric resonances. The 1-D scan of the coplanar lines demonstrates a optimum agreement with the electrostatic FEM simulations, revealing the absence of perturbations to the E-field. Moreover, the fidelity of the spatial scan shows the spatial resolution to be under 20 μm, confirming its relation with the optical mode dimensions within the PhC. This spatial resolution limit goes beyond the limits up-to-date based on similar technologies. In order to complete this work, a comparison of the performances of the EO photonic crystal sensor with sensors based on EO waveguides^51–54^ and bulk EO crystals^55–59^ has been performed. Table 1 summarizes the achieved permormances of each configuration.

**Table 1:** Comparison of the properites and performances of different EO-based electric field sensors. In bold we show the performances achieves in this work.

Properties\Tranducer	EO photonic crystal	EO optical waveguide	Bulk EO crystal
Spatial resolution (m^3^)	**10**^**−5**^ × **10**^**−5**^ × **0**.**7 10**^**−6**^	10^−5^ × 10^−5^ × 10^−2^	0.5 10^−3^ × 0.5 10^−3^ × 10^−3^
Min. E-field (V.m^−1^.Hz^−1/2^)	**32**	1–10	0.15
Frequency bandwidth (Hz)	**10**^**12**^ (**theoretical**)	5 10^9^	4 10^10^
Selectivity (dB)	**>20**	>15	>40
Dynamic range (dB)	**>100**	>100	>100
Invasiveness	**Weak**	moderate	moderate

## Conclusion

The results shown here demonstrate the advantages of a GRs-based reflection Lab-on-fiber sensor for E-field detection. Its ultra-compact size, added to the all-dielectric composition, makes it suitable for applications where the distortion of the E-field is critical, in presence of intense fields or when EM-invisible or safe probe, non-inductive, are required. However, this probe is extremelly suitable when high spatial resolution and large bandwidth performances are required being interesting for applications in printed circuit board (PCB) industry where electric components tend to be more compact and the signals, higher in frequency^60^. The Fano fibered-sensor probe may go beyond the limits that the actual probes present. In addition, the probe can also fulfill the needs of two recent cutting-edge technologies, known as Microwave Therapy and Irreversible Electroploration (IRE) technique, which are giving promising results for the treatment of cancer^61–63^. These techniques use highly localized E-fields for the ablation of tumoral cells which requires ultra-compact and high resolution probes to monitor the applied E-field distribution on the tissue in order to avoid side effects. Moreover, recently PCS have been demonstrated to act as quasi-perfect absorbers for THz waves due to its ability to trap these short wavelenghts^64^. This has been employed to trap the waves into a horn antenna, increasing its gain. This trapping feature can be combined to the ultra-short cavity length of the Fano probe to efficiently detect the THz waves by the intensity modulation of an optical carrier, overcoming the limitations of this emerging technology.

## Methods

### Simulation of the PCS located on the fiber facet

The simulation window is surrounded by perfectly matched layer (PML) media on the three dimensions to inhibit parasitical reflections on the borders. The spatial step along the in-plane directions has been set uniform with a value of 30 *nm*. Meanwhile, in the z-direction a non-uniform distribution is set, which describes a higher resolution within the PCS than in the air surroundings in order to optimize the required computer resources. The single mode fiber is introduced into the simulation window employed for modelling the free-standing PCS. This fiber is defined as step-index single mode and characterized by the refractive index of the cladding (*n*_*clad*_ = 1.4468) and of the core (*n*_*core*_ = 1.4521). The space between the fiber facet and the membrane is defined as air (*n* = 1) with a length *d*. The transmission is computed by appraising the Poynting vector flux through a detector plane which corresponds to the cross-section of the fiber core. The collected energy is normalized with respect to the incident signal. The reflected light is collected at a detector plane placed on the side of the source. The source is set within the fiber and its spatial distribution is described by the fundamental guided mode (*HE*_11_ mode) of the fiber computed by beam propagation methods (BPM).

### Integration of the photonic structure onto the fiber facet

The point welding method relies on the inducted deposition of Pt-based weldings around the membrane in order to bond it to the fiber facet. The membrane shape is chosen to be an octogone inscribed within a circle with a radius slightly smaller than the section of a SMF fiber. This orthogonal shape and its extended size of the membrane avoids pollution during the deposition within the sensing area, remaining most of the residual Pt nearby the membrane borders. We employ a conductive layer based on Cr, deposited by sputtering, with a thickness of 200 *nm* (much higher than the Ga+ ion implantation depth since Cr is also milled during the exposure) in order to protect the LN from the Ga+ ions implantation, which has been reported to produce a depth of 75 *nm* for 30 *keV*^65^. The intrinsic stress of the sputtered layer is studied and controlled to avoid any induced bending within the 700 *nm*-thick TFLN membrane. On the other side, the fiber is covered by a sputtered 20 *nm*-thick layer of Cr in order to provide electrical conductivity which avoids electrical forces during the assembly. Then, the fiber is cleaved, ensuring the lack of metal at the facet. We have observed that the charge effect remains during the SEM imaging, however it is not strong enough to produce any motion since the fiber sidewalls are still metallized. Furthermore, the stress elements of the PM fiber can be observed at the Scanning Electron Microscope (SEM), allowing the alignment operation of the PhC and the LN crystallographic axes with the PM axes to be performed within the dual beam chamber. Once the welding is completed, the micromanipulator tip is milled in order to release the sensor.

### Design of the coplanar electrodes

Three coplanar lines are designed on a Borofloat 33 (BF33) substrate which, due to its transparency at VIS range, provides favorable conditions for the measurement of the distance between the fiber tip and the electrodes. The fabrication of the electrodes is performed through lift-off techniques based on inversible photoresists. The Ti-09 resist is spin coated and isolated. The isolation dose and the development time have been optimized with the aim to maximize the inverted flancs. Then, a thin film of 100 *nm*-thick Au thin layer is deposited by evaporation, followed by the stripping performed by bathing it into remover 1165.
